# The role of prior probability in forensic assessments

**DOI:** 10.3389/fgene.2013.00220

**Published:** 2013-10-28

**Authors:** William C. Thompson, Joëlle Vuille, Alex Biedermann, Franco Taroni

**Affiliations:** ^1^Department of Criminology, Law and Society and School of Law, University of California, IrvineIrvine, CA, USA; ^2^Department of Criminology, Law and Society, University of California, IrvineIrvine, CA, USA; ^3^Faculty of Law and Criminal Justice, School of Criminal Justice, Institute of Forensic Science, University of LausanneLausanne, Switzerland; ^4^Department of Economics, Università Ca' Foscari VeneziaVenice, Italy

**Keywords:** DNA, Bayes Theorem, prior probability, expert testimony, forensic science

As the importance of forensic science in the legal system has grown, debate has arisen about the way forensic scientists should characterize their findings in order to communicate most effectively with legal fact-finders. This article will focus on one aspect of that debate: the framing of conclusions involving elements of probability. In particular, we will examine the contentious issue of whether forensic scientists, when asked to provide evidence that will be used to evaluate various competing propositions about physical evidence, should consider the prior probabilities that those propositions are true. Disputes about this issue have arisen in a number of contexts and recent examples suggest that opinions still diverge (e.g., Budowle et al., 2011; Biedermann et al., 2012). In this comment, we will argue that a reasoned approach to this issue depends on the role that forensic scientists are expected to play in the legal system.

To illustrate the underlying issues, let us begin with a generic example. A forensic scientist is asked to perform DNA profiling analyses of blood found at a crime scene and to compare the result to the DNA profile of a defendant who is charged with the crime. The defendant's guilt or innocence will be determined by a jury. The jurors' decision will depend in part on *their* assessment of two propositions of interest—H1: that the defendant was the source of the blood; and H2: that someone else was the source of the blood. What should the forensic scientist tell the jurors about the results of the DNA analysis?

The jurors might want the expert to tell them definitively which hypothesis is true, or to give them particular values for the so-called source probabilities—saying, for example, that there is a 0.998 probability the defendant is the source of the blood and only a probability of 0.002 that someone else was the source. But there is no way for the forensic scientist to reach such conclusions based on the forensic findings alone. To assess source probabilities, the forensic scientist must also consider other evidence in the case.

Suppose, for example, that the expert found that the defendant and the blood from the crime scene share a set of genetic markers found in one person in 1 million in the relevant population. Without considering other evidence in the case, the expert might make statements about the conditional probability of finding these results under the two hypotheses of interest. For example, the expert might conclude that the shared genetic markers were virtually certain to be found under H1 (defendant was the source), but had only 1 chance in 1 million of being found under H2 (someone else was the source). Based on this assessment the expert might also provide to the jury a so-called likelihood ratio—saying, for example, that the DNA profiling results are 1 million times more probable if the defendant rather than some other person was the source of the blood. But a likelihood ratio is not the same thing as a source probability. The likelihood ratio reflects the relative probability of the findings under the relevant propositions, not the probability that the propositions are true.

The only coherent way to draw conclusions about source probabilities on the basis of forensic evidence is to apply Bayes' rule, which requires that one begins with an assignment of prior probabilities to the propositions of interest (e.g., Robertson and Vignaux, 1995; Finkelstein and Fairley, 1970). Bayes' rule specifies how one ought to combine prior probabilities with the results of a DNA profiling analysis in order to find the so-called posterior probabilities that the defendant is the source of the blood. But the Bayesian approach will only work if the expert can begin with a prior probability.

This brings us to the crux of the debate: whether forensic scientists should even try to specify prior probabilities and, if so, how. It is occasionally suggested that forensic scientists should *assume* equal prior probabilities. This is sometimes described as a position of neutrality and is often justified with references to vague accessory “principles,” such as the “Principle of Indifference” or the “Principle of Maximum Entropy,” borrowed from other disciplines and contexts (Biedermann et al., 2007).

A prominent illustration can be found in paternity cases. When DNA analysts are asked to assist in the assessment of whether a particular man is the father of a child, they usually analyze the profiles of the mother, child, and the accused man, and assign conditional probabilities that the genetic characteristics found in the child (Ec) would be observed under two relevant hypotheses specifying that the accused is the father (H1) and that some other man (from a particular reference population) is the father (H2) conditioned on the alleged parents' DNA profiles (Em and Eam, for the mother and the accused man, respectively). In some cases, the analysts limit themselves to reporting the ratio of these conditional probabilities—i.e., Pr(Ec|Em,Eam,H1)/Pr(Ec|Em,H2)—which is a likelihood ratio (although it is also referred to as the paternity index). But quite often, analysts go farther. They assume that the prior odds of H1 and H2 are equal and then, in accordance with Bayes' rule, they multiply the prior odds by the likelihood ratio (paternity index) to determine the posterior odds of paternity. Recall that odds are defined as a ratio between two probabilities; in this particular scenario, it is the ratio between Pr(H1) and Pr(H2). The posterior odds are typically restated as a probability. For example, if the DNA evidence supports paternity with a likelihood ratio of 1 million some analysts would report a probability of 0.999999 that the accused is the father.

While this approach is commonly used in civil paternity cases, courts in the United States have generally not allowed analysts to characterize their findings in this manner when paternity tests are offered as evidence in criminal cases—e.g., to prove the defendant committed rape or incest by showing he fathered a particular child. The assumption of equal prior odds appears to conflict with the presumption of innocence to which defendants in criminal trials have traditionally been entitled. In the view of most commentators, assuming that the accused starts with a probability of guilt of 0.5 falls far short of presuming him innocent. More fundamentally, making *any* default assumption about the prior probability is seen as violating the obligation of the legal system to deliver individualized justice based on the facts of each case (the attentive reader might have noted that circumstantial information *I* was omitted from the above mathematical notation). Consider that an assumption of equal priors is applied regardless of any other evidence in the case: an accused man who offers proof that he is infertile due to azoospermia and was not on the same planet as the mother at time of conception (i.e. an azoospermic cosmonaut) is treated the same as any other man. While the jury can take the other evidence into account they may have difficulty integrating it with the “probability of paternity” delivered by the forensic expert, or they may mistakenly assume that the “probability of paternity” is all they need consider.

Another suggested approach is that forensic scientists take upon themselves the responsibility for assessing the prior probability of the relevant hypotheses before updating them based on the scientific findings in accordance with Bayes' rule. For example, in the context of missing person identification, commentators declared that “[t]he forensic DNA community needs to develop guidelines for objectively computing prior odds” (Budowle et al., 2011, p. 15). The major objection to this approach, in the context of a criminal trial, is that it may result in forensic scientists going beyond their scientific expertise and usurping the role of the fact-finder. In order to assign prior contextually meaningful probabilities, the expert would need to take into account all of the evidence in the case. But experts are rarely in a good position to evaluate the non-scientific evidence and have no business doing so. The legal system places the responsibility for evaluating the evidence in a case on the fact-finder, whether judge or jury, not the expert witness. Jurors are carefully chosen for the task, are often shielded by evidentiary rules from information that the legal system determines that they should not consider, and are carefully instructed on the presumptions to make and standards to apply in reaching a verdict; experts are not. Allowing expert witnesses to take into account prior odds when considering the probative value of a scientific observation also raises the danger of double-counting certain pieces of evidence (Thompson, 2011).

Consequently, many commentators have suggested that forensic experts have no role in assessing prior probabilities. Because posterior probabilities can only be arrived at by assessing prior probabilities, they argue that experts cannot legitimately make statements about posterior probabilities either. As Redmayne explains (2001, p. 46): “(…) *the expert should not testify in terms such as (…) ’the blood probably came from the defendant', because one can only reach conclusions of this sort by making assumptions about the strength of other evidence against the defendant.”*

There may, however, be circumstances in which a forensic scientist could appropriately assign prior probabilities and use them as a basis for reaching other conclusions. One such circumstance arises when the expert is given the responsibility of making an overall evaluation of a case. For example, coroners are sometimes given full responsibility for determining the cause and manner of a death for legal purpose. (In jurisdictions of the Anglo-Saxon tradition, a coroner is a government official who investigates human deaths and makes independent determinations as to their time, manner, and cause. He should not be confused with the medical examiner, who merely provides information to a court in the course of criminal prosecution or civil litigation but has no judicial authority of his own). In such cases, the expert should certainly take account of all relevant evidence, including both scientific and non-scientific factors. There is no danger of the expert usurping the fact-finder when the expert *is* the fact-finder. The matter becomes more complicated, however, when an expert who has made a determination in the role of fact-finder is subsequently asked to present evidence to another fact-finder, as when a coroner who has determined that a death was due to homicide rather than suicide in an inquest is asked to testify in a subsequent criminal trial. In such cases, the dangers of usurpation and double-counting of evidence discussed above may still loom large.

Whether forensic scientists should take account of the prior probability of the hypotheses they are asked to help evaluate is a complicated question. The answer depends on the role the forensic scientist will be playing in the legal system. If forensic scientists will make the ultimate determination, for legal purposes, with regard to a particular proposition of interest, then they should, and indeed must, consider their prior probabilities that the hypotheses are true. If, however, the truth of the hypotheses will be addressed by someone else—e.g., a judge or jury—and the forensic scientists' role is limited to providing expert assistance, then forensic scientists should generally confine themselves to assign the conditional probability of the scientific findings under the given hypotheses of interest, and should leave to the legal decision maker the task of assessing prior and posterior probabilities.
